# Pyramiding of *cry* toxins and methanol producing genes to increase insect resistance in cotton

**DOI:** 10.1080/21645698.2021.1944013

**Published:** 2021-06-30

**Authors:** Abdul Razzaq, Arfan Ali, Muhammad Mubashar Zafar, Aisha Nawaz, Deng Xiaoying, Li Pengtao, Ge Qun, Muhammad Ashraf, Maozhi Ren, Wankui Gong, Yuan Youlu

**Affiliations:** a State Key Laboratory of Cotton Biology, Key Laboratory of Biological and Genetic Breeding of Cotton, the Ministry of Agriculture, Institute of Cotton Research, Chinese Academy of Agricultural Science, Anyang, Henan, China; b Institute of Molecular Biology and Biotechnology, the University of Lahore, Lahore-Pakistan; c FB Genetics, Four Brothers Group,Lahore-Pakistan; d Lahore College for Women University, Lahore-Pakistan; e School of Biotechnology and Food Engineering, Anyang Institute of Technology, AnyangHenan, China; f University of Agriculture Faisalabad, Faisalabad-Pakistan

**Keywords:** Cotton, transformation, eagle-2, *agrobacterium*, pectin methyl esterase enzyme, insecticidal cry proteins

## Abstract

The idea of enhanced methanol production from cell wall by pectin methyl esterase enzymes (PME) combined with expression of *cry* genes from *Bacillus thuringiensis* as a strategy to improve insect pest control in cotton is presented. We constructed a cassette containing two *cry* genes (*cry1Fa* and *Cry32Aa*) and two *pme* genes, one from *Arabidopsis thaliana* (*AtPME*), and other from *Aspergillus. niger* (*AnPME*) in pCAMBIA1301 plant expression vector using CAMV-35S promoter. This construction was transformed in Eagle-2 cotton variety by using shoot apex-cut *Agrobacterium*-mediated transformation. Expression of *cry* genes and *pme* genes was confirmed by qPCR. Methanol production was measured in control and in the *cry* and *pme* transformed plants showing methanol production only in transformed plants, in contrast to the non-transgenic cotton plants. Finally, insect bioassays performed with transgenic plants expressing *cry* and *pme* genes showed 100% mortality for *Helicoverpa armigera* (cotton bollworm) larvae, 70% mortality for *Pectinophora gossypiella* (pink bollworm) larvae and 95% mortality of *Earias fabia*, (spotted bollworm) larvae, that was higher than the transgenic plants expressing only *cry* genes that showed 84%, 49% and 79% mortality, respectively. These results demonstrate that Bt. *cry*-genes coupled with *pme* genes are an effective strategy to improve the control of different insect pests.

## 1. Introduction

*Gossypium hirsutum L*. is an important economical crop and one of the largest sources of natural fiber worldwide.^1^ Cotton crop is subjected to various biotic and abiotic stresses throughout its life cycle.^2^ However, the biotic stress caused by pathogens and pests has an important negative impact not only on yield and quality, but also in control measures that increases production cost globally.^1^ Cotton fields are prone for lepidopteran infestation such as pink bollworm (*Pectinophora gossypiella*), army bollworm (*Spodoptera litura*), American bollworm (*Heliothis armigera*) and spotted bollworm (*Earias fabia*). The use of chemical insecticides is not an adequate solution since they seriously damage the environment and human health.^3^ *Bacillus thuringiensis* (Bt) is a soil-born bacterium that produces different insecticidal proteins (named δ-endotoxins, such as Cry toxins), which have been successfully used against insect pest attacks and several Cry insecticidal proteins have been also transformed into cotton crops since 1996.^4^ The effectiveness of Bt δ-endotoxins started to decline due to the development of resistance by the insect pests.^5^ Despite the proven substantial protective effects of transgenic Bt-cotton plants against insect attack, improvement is still needed in this technology,^6^ for instance by combining with some enzymes involved in defense strategies against insect pests.^7^ The overproduction of enzymes, involved in insect defense, can be a good alternative to reduce the pest attack and development of insect resistance to Cry toxins.^7^

Plant cell walls are heterogeneous structures containing cellulose, hemicellulose, pectin, phenolic compounds and cell wall proteins. Pectins are integral components of plant primary cell wall acting as barriers to insect pests. Pectin methyl esterase enzyme (PME) catalyzes desterification of pectin into pectate and methanol in the plant cell wall to regulate an inhibitory response against insect pests.^3,8^ Multiple mechanisms are involved in regulation of PME activity and methanol production in plants such as cell wall pH modifications, expression of inhibitory proteins and differential isoform expression in different tissues at different stages.^9^

In this work, PME from *Arabidopsis thaliana* (*AtPME*, accession no. NP 566842) and *Aspergillus niger* (*AnPME*, accession no. XM_001390469) were used for their overexpression coupled with insecticidal *cry* genes in transgenic cotton and toxicity was evaluated against different Lepidopteran insect pests. We selected to work with the PME isolated from the fungus *A. niger* shows high methanol production in tobacco cell suspension culture.^10^ It is also reported that activity of PME is elicited by the attack of herbivores in different plant species,^7,11,12^ inducing the production of toxic methanol.^13^ Methanol is toxic to insect pests,^14^ damaged and wounded leaves by the insect attack as a primary source of methanol production by the preexisting PME in the cell wall.^15^

## 2. Materials and Methods

### 2.1 Plant Materials

*Gossypium hirsutum* L. Eagle-2 variety was selected for the expression of two *cry* genes (*cry1Fa and cry32Aa*) and two methanol producing genes (*AtPME* and *AnPME*). Eagle-2 seeds were obtained from Four Brothers Seeds Multan-Pakistan and planted at research farm of Four Brothers Lahore-Pakistan.

### 2.2 Sequence Selection and Plasmid Construction

**Figure 1.**
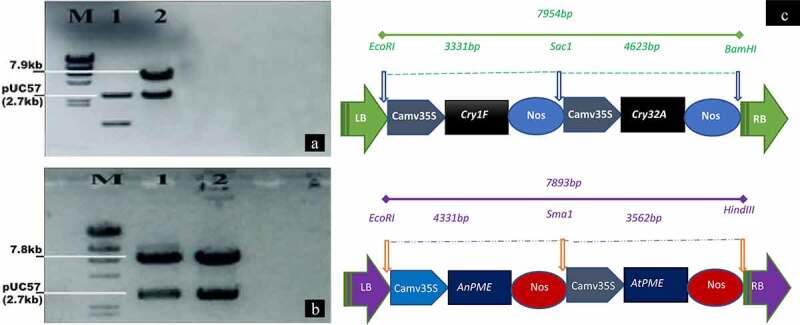
Restriction analysis of puc57 vectors containing the *cry1Fa* and *cry32Aa* gene cassette (total 7954 bp size) or the *AtPME* and *AnPME* gene cassette (total 7893 bp size). Figure 1a, *BamHI* and *EcoRI* restriction analysis of the *cry1Fa* and *cry32Aa* gene cassette. M: 10kb ladder. Lane 2: *cry1Fa* and *cry32Aa* gene cassette positive sample. Lane 1: negative control of pUC57 without insert samples. Figure 1b, *EcoRI* and *HindIII* restriction analysis of the *AtPME* and *AnPME* gene cassette. M: 10 Kb ladder. Lane 1, 2: *AtPME* and *AnPME* gene cassette positive samples. Figure 1c, schematic representation of both gene cassettes containing *cry1Fa* and *cry32Aa* genes or *AtPME* and *AnPME* genes, respectively

Gene sequences of selected *pme* genes (*AtPME*, accession no. NP 566842; *AnPME*, accession no. XM_001390469) were taken from NCBI and after codon optimization, were synthesized by BioBasic, Canada. The synthetic double gene *AnPME, AtPME* cassette (total 7893 bp) was cloned into the *EcoRI* and *HindIII* restriction sites of puc57 vector. While the *cry1Fa, cry32Aa* gene cassette (total 7954 bp) was cloned into the *EcoRI* and *BamHI* restriction sites of pUC57 vector. All genes were under regulation of *CaMV35S* promoter and *Nos* terminator was added at the end of these genes (Fig. 1).

### 2.3 Cloning of Genes into Plant Expression Vector pCAMBIA1301

The two pUC57 vectors with the *AtPME, AnPME* cassette, or the *cry1Fa, cry32Aa* cassette were transformed into *Escherichia coli* top10 competent cells through heat shock method and selected on LB media supplemented with tetracycline (50 µg/ml) and ampicillin (50 µg/ml). Plasmids were isolated by using Gene Jet plasmid DNA isolation kit (Thermo Scientific, Vilnius, Lithuania, Cat#K0503) as indicated in the manufacturer protocol. The presence of these genes was confirmed through restriction digestion using *EcoRI* and *HindIII* or using *EcoRI* and *BamHI* enzymes accordingly to each plasmid. The 7.8 DNA band of *AtPME, AnPME* cassette and 7.9 Kb band of cry*1Fa, and cry32Aa* cassette were visualized in 0.8% of agarose gel electrophoresis and excised fragments were purified by using Gene JET Gel Extraction Kit (Thermo Scientific, Vilnius, Lithuania, Cat#K0503). The purified DNA bands were then ligated to the plant expression vector pCAMBIA1301 pre-digested with the corresponding restriction enzymes and transformed into *E. coli* top10 competent cells. A 1Kb DNA size marker (250bp-10Kb) was received from GeneRuler^TM^ (Thermo Scientific, Cat#SM0311) and used in this study.

### 2.4 Transformation of pCAMBIA1301 vectors with pme and cry gene cassettes into Agrobacterium tumefaciens.

The ligation of the two excised double gene cassettes in pCAMBIA1301 vector was confirmed by restriction digestion using *EcoRI* and *BamHI* enzymes or *EcoRI* and *HindIIII* accordingly to the pCAMBIA1301*-cry*Cassette or to the pCAMBIA1301*-pme*Cassette, and further confirmed by PCR using specific primers (see Table 1 and 4)

**Table 1.** Sequences of the primers used

**Primers**	**Sequence**
GADPH-F	TGATGCCAAG GCTGGAATTGCTT
GADPH-R	GTGTCGGATCAAGTCGATAACACGG
AnPME-F	GGTGCTATCGTTGTTGCTAAGTC
AnPME-R	GCAGTAATTGAAGCAGATGAAGG
AtPME -F	TCTGTTCCTTTGGGTAACACTTG
AtPME -R	GTGATCACGCACCTAAGAAAGAC
Cry1Fa-F	ATGCGCTGTTTACTTCTAGCAAC
Cry1Fa-R	GCACATTTACTGTTTCGTGTTTG
Cry32a-F	CTTTTGCTACGGCTGGACTC
Cry32a-R	TCACTGCCTTTCTGGCTTTT
VirG-F	GAATACCTTACGATCCACGCC
VirG-R	GCGAAACCTGCACGTCCGCG

**Table 2.** Numerical data for transformation experiments in the field

Exp. No.	No. of embryos isolated	Agrobacterium- treated embryos	Embryos on MS plates	Died	Selection tubes	Plantlets died	Plants transferred to pots	plants died in pots	Plants shifted to green house
1	403	403	403	396	7	1	6	1	5
2	345	345	345	341	4	2	2	1	1
3	314	311	311	307	4	2	2	1	1
4	310	305	305	298	7	1	6	3	3
5	350	345	345	340	5	2	3	0	3
6	409	410	410	404	6	2	4	2	2
7	320	319	319	314	5	2	3	1	2
8	410	410	410	404	6	3	3	1	2
9	311	311	311	309	2	1	1	0	1
10	336	335	335	330	5	2	3	1	2
11	400	410	410	406	4	1	3	2	1
12	315	312	312	310	2	1	1	1	0
Total	4223	4216	4216	4159	57	20	37	14	23

**Table 3.** Germination Index

No. of petri plates	Total seeds	No. of germinated seeds	No. of ungerminated seeds	Germination index
1	40	27	21	67.50%
2	40	31

**Table 4.** Transformation efficiency

Agrobacterium treated embryos	Control plants	Plants shifted to green house	transformation efficiency
		Control plants Experimental	Control plants Experimental
4216	50	23 57	46% 1.35%

The optimized PCR conditions used for Cry genes; Initial denaturation at 94°C for 3 minutes, denaturation at 94°C for 45 seconds, annealing at 59°C for 50 seconds, extension at 72°C for 1:30 minutes, final extension at 72°C for 10 minutes whereas the PCR conditions for pme genes; Initial denaturation at 94°C for 3 minutes, denaturation at 94°C for 45 seconds, annealing at 57°C for 45 seconds, extension at 72°C for 1:30 minutes, final extension at 72°C for 10 minutes. The confirmed plasmids were transformed into *A. tumefaciens* strain LBA4404 competent cells by electroporation.^16^ The transformant *A. tumefaciens* cells were grown on YEP media (Peptone 10 g/L, Yeast extract 10 g/L, Sodium chloride 5 g/L, pH 7.5) supplemented with Kanamycin (50 mg/ml) and Rifampicin (50 mg/ml). The appeared colonies of *A. tumefaciens* were then further evaluated through colony PCR as been previously reported^17^ using the specific primers in Table 1.

### 2.5 Agrobacterium Tumefaciens *Mediated Transformation of Cotton Plants*

The seeds of *Gossypium hirsutum* Eagle-2 plant were surface sterilized and placed in the dark at 30°C for 48 h. The germinated seedlings were used for co-transformation using shoot apex cut method. The embryos were isolated, and injury was introduced to the shoot apex before incubation with the Agrobacterium strain LBA4404 harboring the pCAMBIA1301-cry-pme double cassette. The cultures were incubated for 1 hr at 28°C by placing explants on MS solid medium supplemented with (4.4 g/L, Sucrose 30 g/L, Phytagel 2.4 g/L). The embryos were allowed to grow on MS medium plates for 48–72 h supplemented with cefotaxime (500 mg/ml) followed by screening in MS tubes supplemented with hygromycin (25 mg/ml) for one and a half month. After screening, the cotton plants from the tubes were transplanted into pots containing equal proportion of clay, peat moss and sand (1:1:1). Subsequently, the putative transgenic cotton plants were transplanted to the greenhouses of Four Brothers Genetics Inc. for acclimatization and hardening followed by molecular analysis.

### 2.6 Detection of the Two Double Gene Cassettes in Cotton Plants through PCR

Leaves of the putative transgenic cotton plants were harvested, and DNA was isolated for PCR screening of *AnPME, AtPME, cry1Fa*, and *cry32Aa* genes using PCR master mix kit (Thermo Scientific, cat#K1081) using specific primers. In addition, *virG* gene amplification was also done, by using a specific set of primers from the *vir* region, to nullify the *Agrobacterium* contamination. The PCR annealing temperature was set at 60°C.

### 2.7 RNA Extraction and cDNA Preparation

RNA from putative cotton plants was isolated using Agilent kit (Agilent Technologies, Santa Clara, USA, Cat #5185-6000). The RNA was quantified in ng/µl using NanoDrop ND-1000 spectrophotometer at 260 and 280 nm. The DNase-treated total RNA was used to prepare cDNA using the first strand cDNA synthesis kit (Thermo Scientific, Cat #K1632) and cDNA was stored at −20°C.

### 2.8 Expression Analysis of Cotton Transgene

Expression analysis of transgenes was performed by qPCR using specific primers in triplicates with a product size of <200 bp following the protocol of Maxima SYBR Green/ROX (Thermo Scientific, Cat#K0221). The reaction mixture was prepared in a total of 20 µl with the following components of 1 µl of 10 pmol of forward and reverse primers, 5 µl of Maxima® SYBR Green/ROX qPCR Master Mix (2x) and 1 µl (50 ng/µl) of cDNA. Sequences of the primers used for the amplifications of both the genes are given in Table 1. Relative expression was determined according to the 2 ^(-ΔΔCt)^ method using GAPDH primers were used as internal control reference gene for normalization in these qPCR experiments. All assays were done in triplicate.

### 2.9 Methanol Quantification in Transgenic Cotton Plants

Transgenic cotton leaves (1 g) were used for determining methanol concentration. Phosphate buffer was prepared in deuterium oxide composed of 0.03% (w/v) sodium salt of trimethylsilyl propionic acid (TSP) (Sigma-Aldrich). After sonication of the samples, they were centrifuged at 13,000 × g for 10 min and supernatant was collected in a tube for methanol content determination, by using mass spectrometry (MS) with the procedure described by.^10^ Different concentrations of methanol from 0% to 20% were used as standards.

### 2.10 Insect Bioassays

The efficacy of methanol overproduction was tested on insect bioassays against *Helicoverpa armigera, Pectinophora, gossypiella* and *Earias fabia* larvae by comparing toxicity of non-transgenic and transgenic cotton plants. The upper fully expanded positive leaves of the plant that have *H. armigera* and *E. fabia* larvae were removed and placed on moist filter paper in laboratory conditions. A 5–7 larvae of 3^rd^ instar were used per leaf in triplicate. The efficacy of transgenic plants against pink bollworm *(P. gossypiella)* was evaluated by releasing the larvae on young growing bolls and flowers in the field. The mortality rate was observed continuously for 7 days. The mortality rate was calculated by the following formulae;

% Mortality = No. of dead Larvae ∕ Total No. of Larvae × 100

## 3. Results

### 3.1 *Detection of* Pme *or* Cry *Genes Cassettes in pUC57 Vector through Restriction Digestion*

The two plasmids pUC57 that we received from BioBasic, were digested with *BamHI* and *EcoRI* or with *EcoRI* and *HindIII* and resolved on 0.8% agarose gel as shown in Fig. 1a. The expected sizes of 7954 bp and 7893 bp, respectively, for the cassettes containing *cry* genes or *pme* gene cassettes were observed (Fig. 1b). A schematic representation of both gene cassettes containing *cry1Fa* and *cry32Aa* genes or *AtPME* and *AnPME* genes, respectively, is also shown in Fig. 1c.

### 3.2 Detection of Transgene in pCAMBIA through Restriction Digestion Analysis and PCR

**Figure 2.**
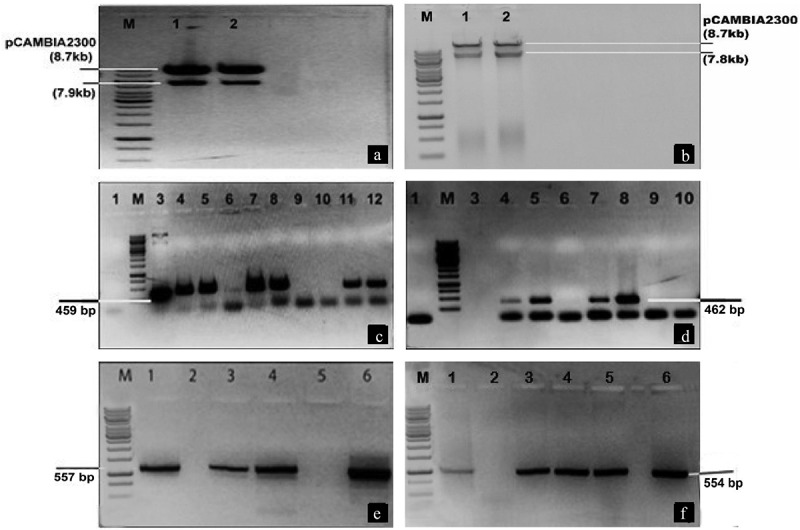
Restriction digestion and PCR analysis of both *cry* genes and *pme* genes cassettes

The purified DNA bands containing these two gene cassettes were ligated to the plant expression vector pCAMBIA1301 pre-digested with the corresponding restriction enzymes. The resulting plasmids were confirmed by restriction digestion analysis. Digestion with *EcoRI* and *HindIII* was done for the plasmid containing *cry* gene cassette and digestion with *EcoRI* and *BamHI* was done for the plasmid containing pme gene cassette. The observed band sizes confirmed their successful ligation in pCAMBIA1301. Both *cry* genes were then amplified resulting in a PCR product of 459 bp for *cry1F* and 462 bp for *cry32A*. Similarly, *AnPME* and *AtPME* genes were amplified through colony PCR resulting in a PCR product of 557 bp and 554 bp, respectively (Fig. 2).

Figure 2a, *EcoRI* and *BamHI* restriction of pCAMBIA1301 vector containing *cry* gene cassette; Lanes 1, 2: show positive samples; Fig. 2b, *EcoRI* and *HindIII* restriction of pCAMBIA1301 vector containing *pme* gene cassette; Lanes 1, 2: show positive samples; Fig. 2c, confirmation of *cry1Fa* gene by PCR, M: 10 Kb ladder; Lane 1: shows negative control; Lane 3: shows positive control; Lanes 4, 5, 7, 8, 11 and 12: show positive samples; Lanes 6, 9, and 10: show negative samples. Figure 2d, confirmation of cry*32Aa* gene by PCR, M: 10Kb ladder; Lane 1, 3: shows negative control; Lanes 4, 5, 7: and 8 show positive samples; Lanes 6, 9, and 10: show negative samples. Figure 2e, confirmation of *AnPME* gene by PCR, M: 1 Kb ladder; Lane 1: shows positive control; Lanes 3, 4, and 6: show positive samples; Lanes 2, and 5: show negative samples. Figure 2f, confirmation of *AtPME* gene by PCR, M: 10 Kb ladder; Lane 1: shows positive control; Lanes 3, 4, 5, and 6: show positive samples; Lane 2: shows a negative sample.

### 3.3 Detection of pCAMBIA1301-PMECassette and pCAMBIA1301-cryCassette constructions transformed into A. tumefaciens

**Figure 3.**
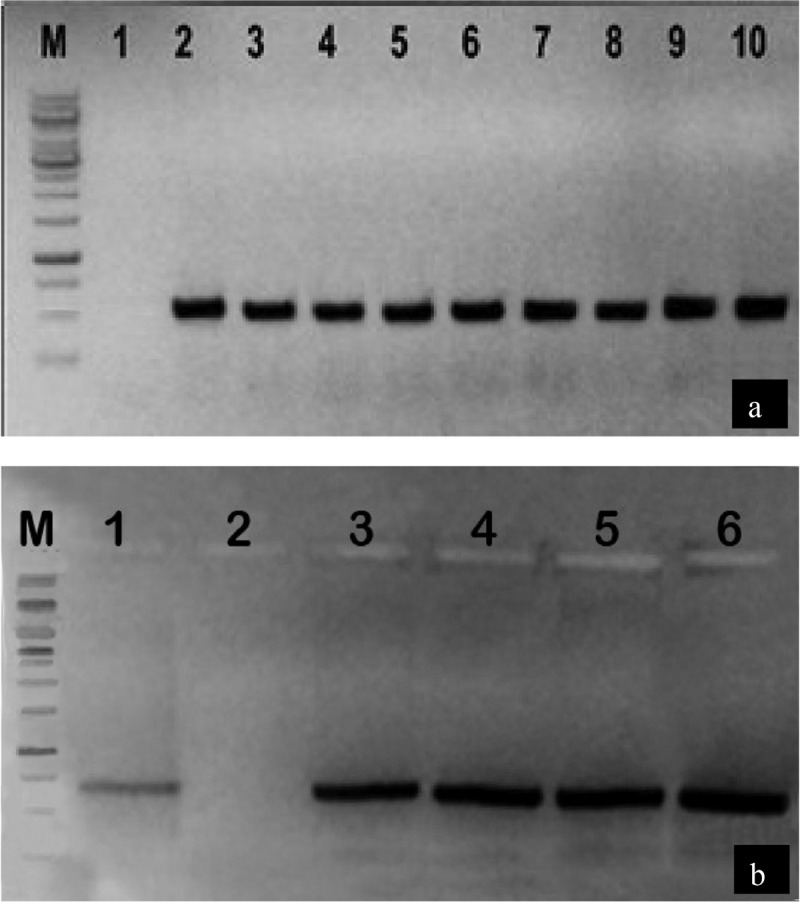
PCR amplification of both *cry* genes and *pme* genes from transformed *A. tumefaciens* with the corresponding pCAMBIA1301-*cryCassette* and pCAMBIA1301-*pmeCassette* constructions

A number of random colonies of electroporated *A. tumefaciens* strain LBA4404 were selected for PCR colony assays, using specific primers designed from *cry1Fa, cry32Aa, AnPME* and *AtPME* genes. The PCR products were resolved on 1.5% agarose gel and our results show that all the colonies amplified the expected PCR product of 459 bp and 462 bp for the *cry1F* and *cry32A, respectively*, and 557 bp and 554 bp for the *Anpme* and *At*pme genes, respectively, with the exception of the negative control (Fig. 3).

Figure 3a, Amplification of both *cry* genes; M: 10 kb ladder; Lane 1: shows negative control; Lane 2: shows positive control; Lanes 3–6: show positive amplification of *cry1Fa*, and Lane 7–10: show positive amplification of cry*32Aa* genes in individual *A. tumefaciens* colonies. Figure 3b, shows an amplification of both *pme* genes, M: 10 Kb ladder; Lane 1: shows positive control; Lane 2: shows negative control; Lanes 3, 4: show positive amplification of *AnPME*, and Lane 5, 6: show positive amplification of *AtPME* genes in individual *A. tumefaciens* colonies.

### 3.4 Transformation of the Two Double Gene Cassettes into Cotton Plants

The seeds of *Gossypium hirsutum* Eagle-2 were sterilized and placed in the dark at 30°C for 48 h. The germinated seedlings were used for transformation using shoot apex cut method. These plants were inoculated with both *A. tumefaciens* strains containing *cry* gene cassette or *pme* gene cassettes. Germination index of Eagle-2 was calculated to be 67.50% whereas transformation efficiency was recorded to be 1.35%.

### 3.5 Detection of Transgenic Cotton Plants

**Figure 4.**
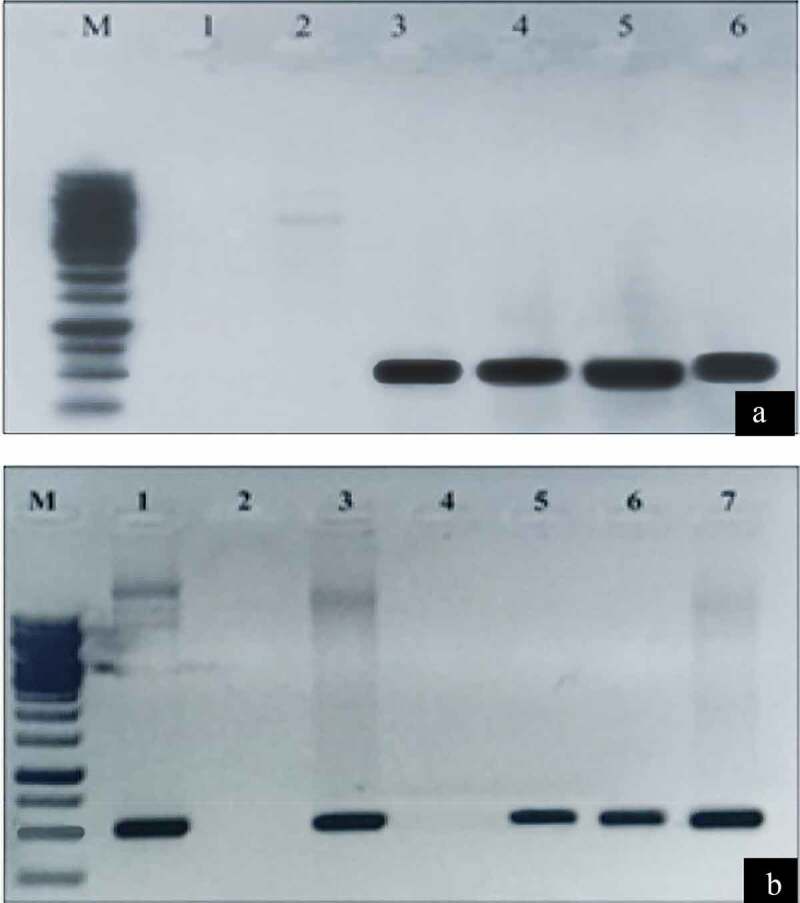
Determination of *cry* genes and *PME* genes expression in transgenic cotton plant by PCR analysis

Fresh leave samples of the transgenic cotton plants were selected for DNA isolation and analysis of successfully transformed *cry* genes and *pme* genes using specific primers that produce PCR products of 459 bp and 462 bp for cry (*cry1F* & *cry32A*) genes whereas 557 bp and 454 bp for *pme* (*Anpme* & *Atpme*) genes, respectively (Fig. 4).

Figure 4a, M: 10 Kb ladder; Lane 1, 2: shows negative sample; Lanes 3, 4: show positive for *cry1Fa* and Lane 5, 6: show positive for *cry32Aa*, positive samples. Figure 4b, M: 10 Kb ladder; Lane 1: shows positive control; Lane 2: shows negative control; Lanes 3, 5: show positive for *AnPME*, and Lane 6, 7: show positive for *AtPME* samples; lane 4: shows negative sample.

**Figure 5.**
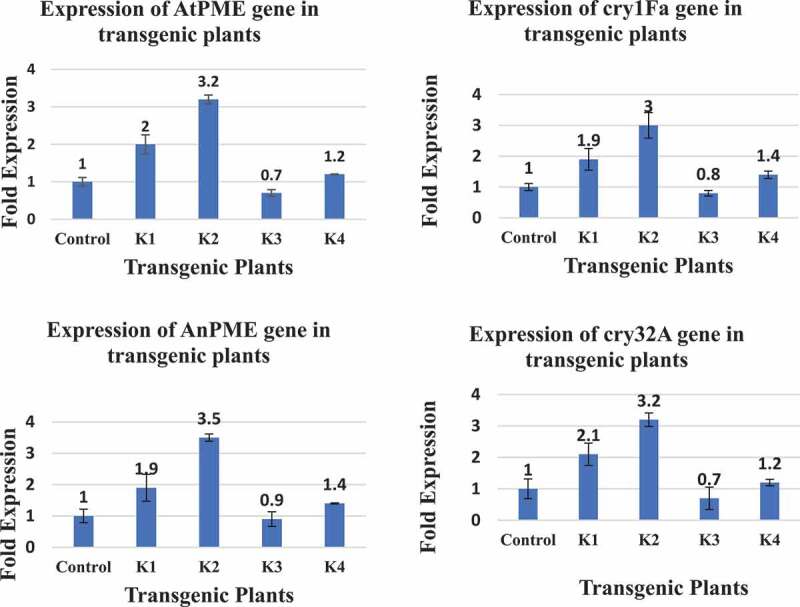
Relative expression of *cry* and *PME* genes in four transgenic cotton plants (K1, K2, K3 and K4)

The mRNA from transgenic plants was isolated and cDNA transcribed. The relative mRNA expression of *cry1Fa, cry32Aa*, AtPME and *AnPME* genes was analyzed by using SYBR Green Mix in qPCR assays and higher expression of these genes was found in transgenic plants (Fig. 5). Expression of *GAPDH* gene was used as internal control reference for normalization in these assays. We analyzed the expression of these genes in four transgenic plants (named K1, K2, K3 and K4) that were positive for transformation with the four genes (*cry1Fa, cry32A, AtPMe* and *AnPME*). Control plants were non-transformed Eagle-2 cotton plants.

The relative expression of *cry* and *PME* analyzed in different plants shown in the figure was calculated according to the 2 ^(-ΔΔCt)^ method using GAPDH as internal control reference gene for normalization.

The K2 plants showed the highest relative highest expression of the four *AtPME* and *AnPME, cry1Fa* and *cry32A* genes, while K3 plants showed the lower expression of these genes.

### 3.6 Evaluation of Methanol Concentration in Transgenic Cotton Plants

**Figure 6.**
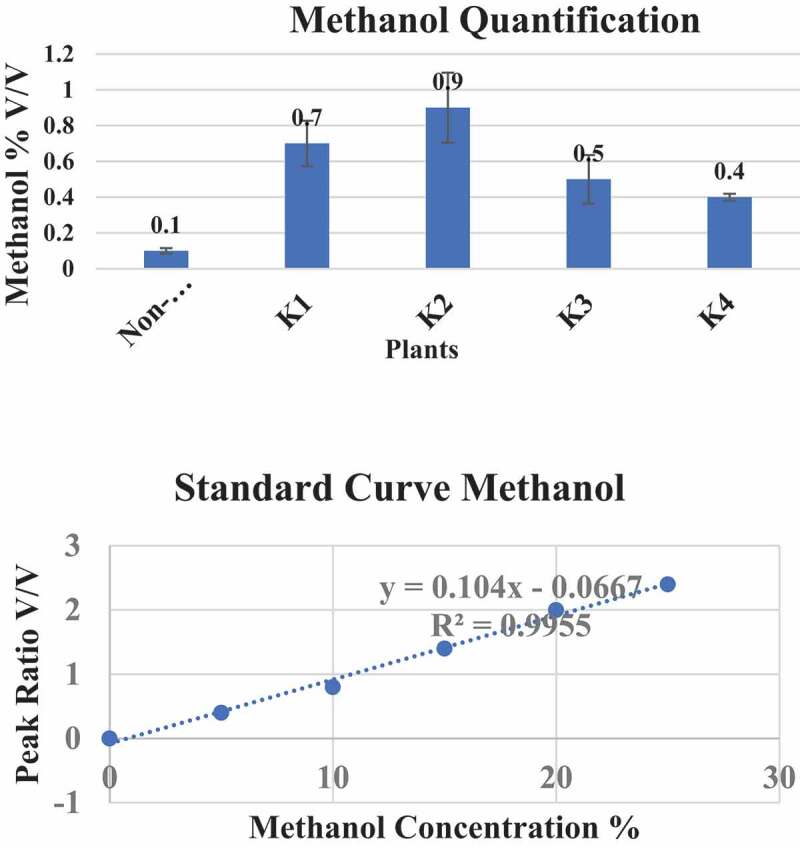
Methanol quantification in transgenic cotton plants. Standard curve of methanol was done by Mass Spectrometry (MS) with reference to a methanol standard. The transgenic cotton plants, namely, K1, K2, K3, K4 were subjected to methanol quantification

Transgenic K1-K4 cotton plants were subjected to Mass Spectrometry (MS) for methanol quantification. Transgenic plants K1 and K2 showed the highest contents of methanol, respectively, as compared to K3 and K4 plants and control plants. These data indicated that the methanol production in transgenic plants showed higher values than non-transgenic cotton plants (see Fig. 6).

### 3.7 Evaluation of Transgenic Cotton Plants for Resistance against Chewing Insects

Bioassays were performed by using fresh leaves of non-transgenic cotton as negative control, and compared with transgenic cotton plants K1 to K4 expressing double Bt-*cry* genes and *pme* genes. We also compared with cotton plants transformed only with the two Bt *cry*-genes carried out in the laboratory. Each of the sampled fresh leaves was infected with *H. armigera* and *P. gossypiella* larvae. A 5–7 larvae of 3^rd^ instar per leaf were used in triplicates.

**Figure 7.**
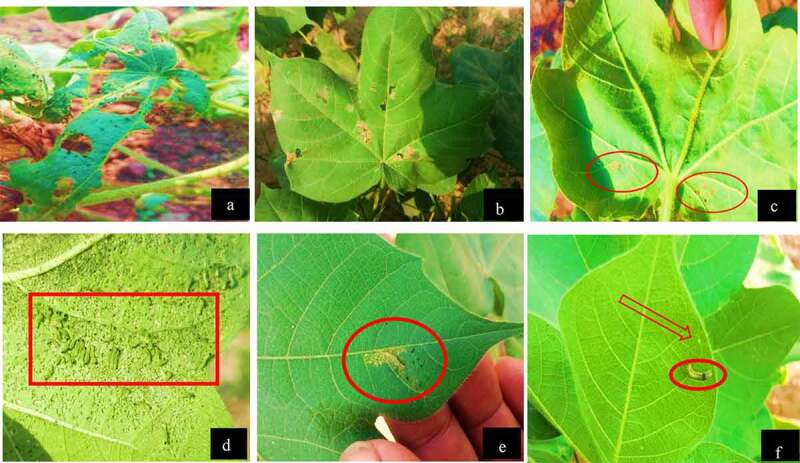
Cotton leaf chewing and mortality assay of *H. armigera* in different transgenic cotton plants

The infestation *H. armigera* data were recorded, showing 100% mortality on the third day after infection of transgenic cotton plants harboring both Bt *cry*-genes coupled with *AtPME* and *AnPME*, while 84% mortality was observed after fifth days in the control plants transformed only with Bt-*cry1Fa* and *cry32* genes and no mortality was observed in negative control of Eagle-2 cotton plants as shown in Fig. 7.

In the case of *P. gossypiella* larvae were released on freshly growing bolls of the cotton plants. Transgenic cotton plants expressing *AtPME* and *AnPME* along with Bt *cry*-genes showed 70% mortality of *P. gossypiella* larvae after 3 days, implying resistance toward *P. gossypiella* larvae. In contrast to transgenic cotton plants harboring only the two Bt *cry*-genes that showed 49% of *P. gossypiella* mortality and 0% mortality was observed in non-transgenic Eagle-2 control plants.

**Figure 8.**
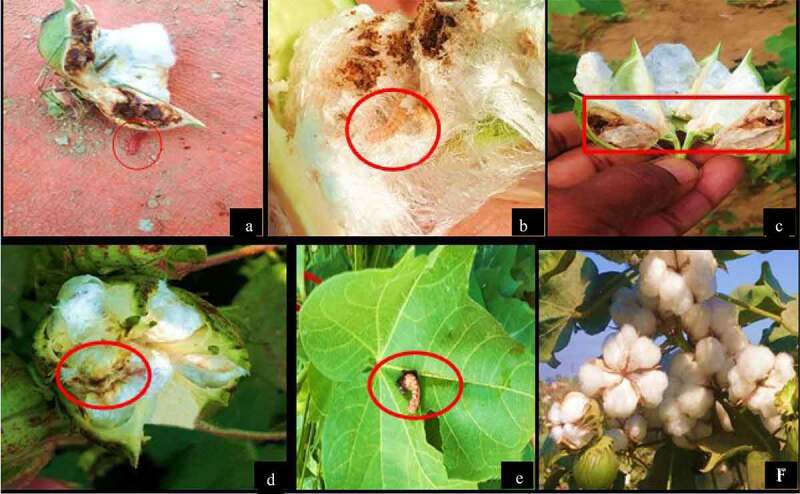
Pink Bollworm *P. gossypiella* mortality assays in different transgenic cotton plants. Figure 8a and b, in non-transgenic cotton plants, the cotton boll showed to be completely damaged by *P. gossypiella* and and larvae that were still alive are highlighted with a red circle mark. Figure 8c and d, in transgenic plants transformed with the double Bt-gene (*cry1Fa* and *cry32Aa*), the plant bolls showed insect resistance and only one locule was damaged but *P. gossypiella* larvae were dead. Figure 8e and f in the transgenic plants transformed with double Bt-gene (*cry1Fa* and *cry32Aa*) and double *AnPME* and *AtPME* gene in the same plant; the plants showed full resistance and even spotted was found dead on leaf and *P. gossypiella* free cotton was fully developed

**Figure 9.**
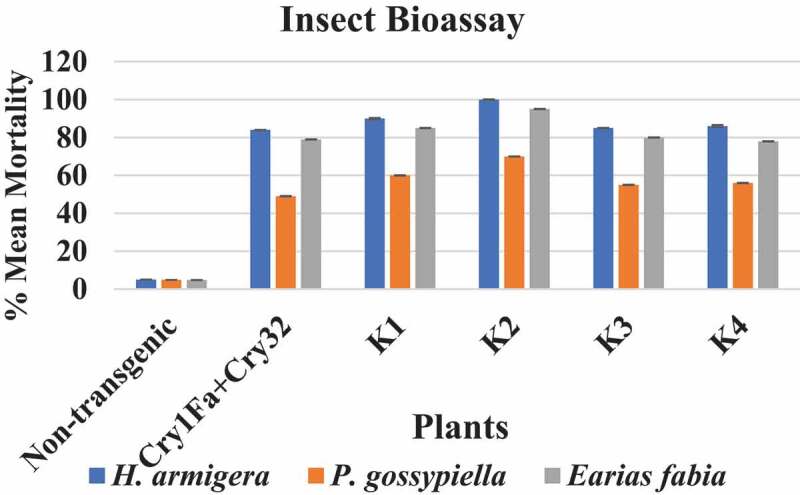
Mortality analysis against army boll worm *Helicoverpa armigera*, Pink boll worm *Pectinophora gossypiella* and spotted bollworm *Earias fabia*. Two-way ANOVA showed the significance of the data. compared to the non-transformed plants

Finally, regarding spotted Bollworm (*Earias fabia*) 95% mortality was observed in transgenic harboring double Bt *cry* and *pme* gene cassettes, while 79% mortality was observed in transgenic plant expression only the double Bt *cry*-genes (Fig. 8). Two-way ANOVA showed the significance of our data at *P*≤ 0.0001 (Fig. 9).

Figure 7a, Non-transgenic cotton plant leaf completely chewed by *H. armigera*. Figure 7b, Double Bt-gene (*cry1Fa* and *cry32Aa*) plants showed resistance and a little bit chewed by *H. armigera*. Figure 7c, Double Bt-gene (*cry1F*a and *cry32Aa*) and double *AnPME* and *AtPME* gene in the same plant showed full resistance and just a minor cut was observed by *H. armigera*. Figure 7d, Hatching of *H. armigera* on negative control Eagle-2 cotton plant leafs. Figure 7e, in double Bt-gene (*cry1Fa* and *cry32Aa*) cotton plant leafs, the *H. armigera* were dead after eating scratched looking portion of leaf. Figure 7f, In the double Bt-gene (*cry1Fa* and *cry32Aa*) and double *AnPME* and *AtPME* gene plants, the *H. armigera* were dead even after a minor first cut.

## 4. Discussion

Cotton is considered a socioeconomically important crop^18–20^ and due to current trend of global market it is necessary to make continuous improvements in cotton varieties.^21^ The transformation of new genes into cotton varieties is required to develop resistance against unrelated invading insect pests.^22^ For this goal, a unique approach was adopted therein two cassettes were designed. One cassette harbor two pectin methyl esterase genes (*pme*) from *A. thaliana* and *As. niger* and second cassette contains two insecticidal proteins coded by *cry* genes from *B. thuringiensis*. Both cassettes were transformed into non-transgenic cotton variety Eagle-2 to achieve resistance against insects as previously depicted in tobacco plants.^8^ Initial screening of putative cotton plants was done by hygromycin as reported.^23^ Putative transgenic cotton plants were confirmed through PCR by using specific primers as previously done.^24^ PCR confirmed transgenic plants were subjected to qPCR for determination of mRNA expression of the transgenic genes as reported.^25^ Ultimately, the efficacy of transgene was determined by insect bioassay against different target Lepidopteran larvae.^26^

Naturally, plants produce methanol, which is nontoxic for the plants.^27^ Methanol after accumulation in leaves is released into atmosphere through stomata^28,29^ and is found to be toxic to the insects.^8^ In this study, the methanol quantification was done in transgenic and non-transgenic plants by MS as reported before.^10^ Transgenic cotton plants K1 and K2 showed a 0.7% and 0.9% increase in methanol concentration when compared with the non-transgenic control plants, while K3 and K4 transformed plants showed only 0.5% and 0.4% methanol concentration, which is less than K1 and K2 plants, but greater than a control plant. Methanol concentration was calculated in correlation with insect bioassay in which K1 and K2 with higher expression of all genes showing 100% mortality in accordance with Hasunuma et al. (2003).

In this study, real-time qPCR assays were conducted to analyze mRNA expression of *pme* and *cry* genes in transgenic cotton plants. The relative expression of *AtPME* and *AnPME* was higher in K2 than in control plants. Insect bioassays were performed on detached leaves, flower and bolls of cotton. The infestation data were recorded and 100% mortality was observed after three days in transgenic cotton plants harboring both Bt *cry*-genes coupled with *AtPME* and *AnPME* genes, while 84% mortality was observed in Bt *cry*-genes transgenic cotton plants after five days, while there was no mortality observed in negative control cotton plants as shown in Fig. 8. The Pink Bollworm (*P. gossypiella*) larvae were released on freshly growing bolls of the cotton plants. Transgenic cotton plants showed resistance toward pink bollworm larvae for 3 days with 70% mortality in case of transgenic cotton plants harboring *AtPME* and *AnPME* along with Bt *cry*-genes in contrast with the 49% mortality observed in transgenic cotton plants harboring only the two Bt *cry*-genes and 0% mortality in non-transgenic control plants. Finally, we observed 95% mortality in spotted bollworm in transgenic plants harboring double Bt-genes and *pme* genes, while only 79% mortality was observed in transgenic plants expressing only the double Bt *cry*-genes as shown in Fig. 9. These results indicated that Bt *cry*-genes coupled with *pme* genes are a possible and useful strategy to control different insect pests and for lowering the resistance of insects against transgenic cotton varieties.

## 5. Conclusion

Transgenic plants expressing simultaneously *pme* and *cry* genes were evaluated against different Lepidopteran insect pests and compared against non-transgenic and transgenic plants expressing only Bt *cry-*genes. Increased mortality in insects was observed in transgenic plants harboring *pme* and *cry* gene combination as compared with positive control expressing *cry* genes only. The increased production of ethanol by *pme* genes in these transgenic plants explains their improvement in the control against insect attack. This control strategy infers that it may be robust to reduce the attack of different lethal cotton insects. As it is reported that insect attack has become a major concern in cotton growing countries around the world especially in Pakistan where farmers have started walking out from the cultivation of cotton due to the high risk of insect attack. Therefore, the proposed strategy may incur some positive results to win the farmer’s interest in the cotton cultivation.

## 6. Compliance with Ethical Standards

### 6.1 Potential Conflict Interest

The authors confirm that there is no actual or potential conflict of interest and also that the work described in this manuscript has not been published previously, that it is not under consideration for publication elsewhere. Its publication has been approved by all authors and implicitly by the responsible authorities where the work was carried out, and all persons entitled to authorship have been so named. The authors also confirm that, if accepted, it will not be published elsewhere including electronically in the same form, in English or in any other language, without the written consent of the copyright-holder.

### 6.2 Research Involvement with Human Participants/ or Animals

The research does not involve with human and animal participants.

### 6.3 Informed Consent

I have read and I understand the provided information and have granted the permission to ask the questions. I understand my participation in the research and liable to produce the given information at any stage.
